# Budesonide and fluticasone propionate differentially affect the airway epithelial barrier

**DOI:** 10.1186/s12931-015-0318-z

**Published:** 2016-01-06

**Authors:** I. H. Heijink, M. R. Jonker, M. de Vries, A. J. M. van Oosterhout, E. Telenga, N. H. T. ten Hacken, D. S. Postma, M. van den Berge

**Affiliations:** Department of Pathology & Medical Biology, Experimental Pulmonology and Inflammation Research, University of Groningen, University Medical Center Groningen,, Hanzeplein 1, NL-9713 GZ, Groningen, The Netherlands; Department of Pulmonology, University of Groningen, University Medical Center Groningen, Groningen, The Netherlands; University of Groningen, University Medical Center Groningen, GRIAC Research Institute, Groningen, The Netherlands

**Keywords:** Bronchial epithelial cells, COPD, Pneumonia, Cigarette smoke extract, Poly-(I:C)

## Abstract

**Background:**

COPD patients have a higher risk of pneumonia when treated with fluticasone propionate (FP) than with placebo, and a lower risk with budesonide (BUD). We hypothesized that BUD and FP differentially affect the mucosal barrier in response to viral infection and/or cigarette smoke.

**Methods:**

We assessed protective effects of equivalent concentrations of BUD and FP on cytokine production and barrier function (electrical resistance) in human bronchial epithelial 16HBE cells and primary bronchial epithelial cells (PBECs) upon exposure to viral mimetic poly-(I:C) and/or cigarette smoke extract (CSE) or epidermal growth factor (EGF).

**Results:**

BUD and FP were equally effective in suppressing poly-(I:C)- and/or CSE-induced IL-8 secretion in 16HBE and PBECs. Poly-(I:C) substantially decreased electrical resistance in 16HBE cells and both BUD and FP fully counteracted this effect. However, FP hardly affected 16HBE barrier dysfunction induced by CSE with/without poly-(I:C), whereas BUD (16 nM) provided full protection, an effect likely mediated by affecting EGFR-downstream target GSK-3β. Similarly, BUD, but not FP, significantly improved CSE-induced barrier dysfunction in PBECs. Finally, BUD, but not FP, exerted a modest but significant protective effect against *Streptococcus Pneumoniae-*induced barrier dysfunction, and BUD, but not FP, prevented cellular adhesion and/or internalization of these bacteria induced by poly-(I:C) in 16HBE.

**Conclusions:**

Collectively, both BUD and FP efficiently control epithelial pro-inflammatory responses and barrier function upon mimicry of viral infection. Of potential clinical relevance, BUD more effectively counteracted CSE-induced barrier dysfunction, reinforcing the epithelial barrier and potentially limiting access of pathogens upon smoking in vivo.

## Background

Chronic Obstructive Pulmonary Disease (COPD) is a chronic inflammatory respiratory disease affecting millions of people worldwide. Inhaled corticosteroids (ICS) are widely used in the management of COPD. ICS effectively reduce the number of exacerbations and improve respiratory symptoms and quality of life [1]. However, ICS use may also increase the risk of pneumonia in COPD [2, 3]. The TORCH study demonstrated this for the first time, comparing fluticasone propionate (FP) and placebo [2, 3]. Findings from this study were confirmed in a meta-analysis by Singh and colleagues [4]. Sixteen of the 18 studies included in the meta-analysis of Singh and colleagues investigated the effects of FP or FP/salmeterol, and it remained unclear whether the increased pneumonia risk would be FP specific or a class effect of ICS and also present with budesonide (BUD) treatment. More recent studies suggested that pneumonia events were lower with BUD than with FP treatment [5, 6]. Furthemore, Suissa and colleagues reported that FP treatment is associated with a substantial increase in the risk of serious pneumonia in COPD patients, while the risk with BUD was comparatively low, even at high doses [7]. Most recently, Suissa and colleagues reported that discontinuation of ICS use in COPD is associated with a reduction in the elevated risk of serious pneumonia, especially for FP [8]. Thus, the increased risk to develop pneumonia in COPD may be specific to the use of FP and not the result of a class effect of ICS. The cellular mechanisms underlying these differences in safety for ICS use in COPD patients are not well understood. BUD is less lipophilic than FP and has a higher aqueous solubility, leading to a shorter retention time in the lining fluid of the airways, while after being absorbed, BUD is retained in airway tissue/epithelium for a longer time than FP [9, 10]. It is as yet unknown how this may affect the action of BUD and FP in epithelial cells.

The bronchial epithelium forms the first continuous physical barrier to microbial infections and is part of the innate immune response, producing antimicrobial and pro-inflammatory peptides/cytokines acting on immune cells, the latter especially when the epithelial layer is damaged. In COPD, aberrant epithelial repair in response to cigarette smoking may disturb epithelial barrier function [11] and we previously observed a reduction in epithelial barrier function upon smoke extract exposure in vitro [12]. Compromised barrier function may render the airways more susceptible to pathogens, and accordingly, rhinovirus-induced barrier dysfunction in mice was shown to increase the risk of a secondary bacterial infection [13]. The corticosteroid dexamethasone improves airway and corneal epithelial barrier function in vitro [14–16]*.*

We hypothesized that BUD is more effective than FP in protecting against airway epithelial barrier dysfunction upon damage by environmental insults. Viral infection may predispose to bacterial pneumonia, activating toll-like receptor 3 (TLR3) on airway epithelium [13, 17]. TLR3-dependent effects have also been demonstrated for *Haemophilus Influenza*, one of the most common causes of pneumonia in COPD [18]. Therefore, we compared the effect of BUD and FP on viral mimetic poly-(I:C) and/or cigarette smoke-induced epithelial barrier function and pro-inflammatory cytokine production in both the human bronchial epithelial cell line 16HBE and cultured primary bronchial epithelial cells (PBECs) of smoking individuals with normal lung function.

## Methods

### Cell culture

The human bronchial epithelial cell line 16HBE was kindly provided by Dr. D.C. Gruenert (University of California, San Francisco, CA) and cultured in EMEM medium/10 % FCS (Biowhittaker, Verviers, Belgium) supplemented with 100 U/ml penicillin and 100 μg/ml streptomycin on collagen-coated flasks as described previously [19]. PBEC cultures were obtained from bronchial brushings in six current smoking individuals with ≥10 pack-years, FEV_1_/FVC > 70 % and FEV_1_ > 90 % of predicted and not using inhaled corticosteroids, long-acting β_2_- adrenergic agonists and long-acting anticholinergics for at least 4 weeks preceding the study. The Medical Ethics Committee of the University Hospital of Groningen approved the study. All subjects gave their written informed consent. For studies in mucociliary differentiated cells, PBECs were obtained by protease digestion from trachea-bronchial tissue of 10 non-COPD donor lungs. The study protocol was consistent with the Research Code of the University Medical Center Groningen (http://www.rug.nl/umcg/ onderzoek/researchcode/index) and national ethical and professional guidelines (“Code of conduct; Dutch federation of biomedical scientific societies”; htttp://www.federa.org). Cells were cultured as described previously [20] in bronchial epithelium growth medium (BEGM, Lonza, Walkersville, MD) on collagen/fibronectin-coated flasks and stored in liquid nitrogen to be used for experiments at later time in passage 3.

### Treatment of the cells

We used BUD and FP in equivalent concentrations with a dose ratio of FP:BUD = 1.6, based on the observations from clinical studies that 800 μg BUD is equivalent to 500 μg FP. Cells were pre-treated with or without BUD or FP for 2 hours and subsequently exposed to vehicle (medium), 5 or 7.5 % CSE [12], poly-I:C (12.5 μg/ml), EGF (10 ng/ml) or GSK-3β inhibitor CT99021 (1 μM) for 1–24 hours.

#### Viral infection in air-liquid interface-cultured epithelial cells

To induce mucociliary differentiation at the air-liquid interface (ALI), the PBECs from non-COPD donors were grown in duplicates on semi-permeable membranes coated with 30 μg/ml collagen 10 μg/ml fibronectin and 10 μg/ml BSA in a 1:1 mixture of DMEM (Lonza) and BEGM supplemented with retinoic acid (RA, 15 ng/ml; Sigma, St. Louis, MO, USA) and exposed to air for 4 weeks as described previously [20]. Cells were hormonally deprived overnight. For infection with live rhinovirus (RV; major receptor group RV-16, kind gift of D. Davies, University of Southampton, UK), cells in an identically seeded well were counted to calculate multiplicity of infection (MOI) and the virus concentration was adjusted to the number of cells. The apical surface was infected with 50 μl RV16 with an MOI of 1 for 24 hours at 37 °C before harvesting for RNA isolation and collection of supernatants.

#### Exposure to Streptococcus pneumoniae

For bacterial infection, *Streptococcus pneumoniae* strain TIGR4∆cps was used. *S. pneumoniae* was grown in M17 broth (Oxoid,Hamshire, UK) supplemented with 0.5 % glucose, or on blood agar plates (Mediaproducts bv, Groningen, The Netherlands) as described previously [21]. For start inoculations in all experiments, *S. pneumoniae* aliquots were made by growing *S. pneumoniae* in M17 supplemented with glucose to a 600 nm optical density of ~0.25, mixed to a 10 % glycerol concentration and then frozen in 1 ml aliquots at −80 °C. Prior to infection, confluent 16HBE cell monolayers in uncoated transwell plates (Transwell, 3 μm pore-size, 6.5 mm diameter; Costar #3472, Costar Corning Inc., Cambridge, MA) were incubated for 2 hour in infection assay medium, with and without 16 nM BUD and 10 nM FP. Subsequently, ~5*10^6^ CFU of *S. pneumoniae* were added per well and incubated for 2–24 hours, in the presence and absence of 12.5 μg poly-(I:C). To assess *S. pneumoniae* adhesion/internalization, 16HBE cells were washed with PBS/0.01 % CaCl_2_ and subsequently lysed with PBS/0.1 % Triton. Colony forming units (CFUs) were determined by plating serial dilutions on blood agar plates. For analysis of transmigration, the medium was removed from the basolateral compartment after 2, 4 or 24 hours and plated for CFU determination. Before and 24 hours after bacterial infection, transepithelial resistance was measured using a volt-ohmmeter (EVOM, world precision instruments, Sarasota, FL).

### Preparation of cigarette smoke extract

Cigarette smoke extract (CSE) was prepared as described previously [22]. In short, Kentucky 3R4F research-reference cigarettes (The Tobacco Research Institute, Lexington, KY) were used without filter. Smoke from two cigarettes was bubbled through 25 ml medium (100 % CSE). The extract was prepared freshly.

### Electric Cell-surface Impedance Sensing (ECIS)

Electrical resistance of submerged cultured cells was measured using ECIS (Applied Biophysics, Troy, NY) as described previously [23, 24]. Resistance and capacitance were measured at 400 Hz and 40 kHz, respectively. In the ECIS system, all established resistance values were between 10,000-20,000 Ω in the 16HBE cultures and ~1,500 Ω in the primary cell cultures.

### Western blotting

Total cell lysates were obtained and subjected to western blotting using antibodies against E-cadherin, phospho-EGF receptor (EGFR), actin, GAPDH (Santa Cruz Biotechnology, Santa Cruz, CA), zona occludens (ZO)-1 (Invitrogen, Carlsbad, CA, USA) and phospho-GSK-3β (Cell Signalling Technology, Herts, UK) as described previously [25]. Protein levels were quantified using the gelscan program QuantityOne.

### Immunofluorescent staining of ZO-1

Cells grown on LabTeks were washed with PBS/CaCl_2_, fixed in ice-cold acetone (90 %) for 30 min, blocked in PBS/5 % BSA for 60 min, incubated for 60 min with primary antibodies (1:200) against ZO-1 (Invitrogen) and subsequently incubated for 60 min with FITC-labeled anti-rabbit (1:200, DAKO, Glostrup, Denmark) or Rhodamine-labeled anti-mouse IgG conjugates (1:400, Jackson Immunoresearch Laboratories, West Grove, PA).

### Measurement of gene expression with qPCR

RNA was isolated from 16HBE and cDNA synthesized as described previously [26]. We analyzed the expression of E-cadherin and the housekeeping genes PPIA and β2μG. Analyses were performed by real-time PCR using Taqman according to manufacturer’s guidelines using validated probes and the TaqMan Master (Applied Biosystems, Foster City, CA).

### Measurement of IL-8 levels

Protein levels were measured in cell-free supernatants using ELISA kits according to manufacturer’s guidelines (R&D systems Europe Ltd., Abingdon, UK).

### Statistics

Data were analyzed using the paired Student’s t-test, the Wilcoxon-signed rank test or repeated measures ANOVA for ECIS experiments as indicated. Differences were considered statistically significant at p < 0.05.

## Results

### Effects of BUD and FP on cytokine release in 16HBE cells and PBECs

**Fig. 1:**
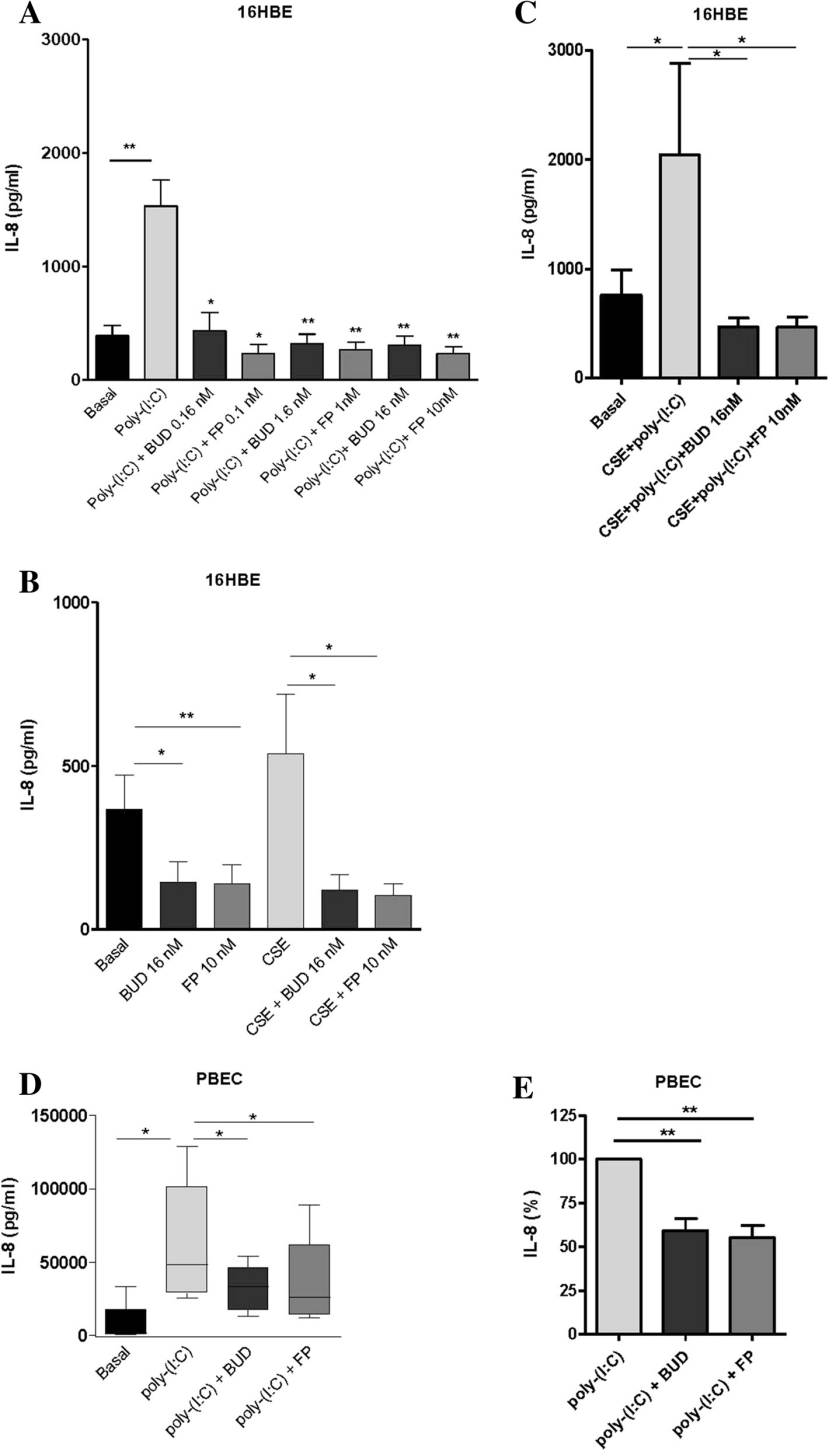
Effect of equivalent concentrations of budesonide (*BUD*) and fluticasone propionate (*FP*) on cigarette smoke extract (*CSE*) and/or poly-(I:C)-induced IL-8 (CXCL8) release in 16HBE cells and primary bronchial epithelial cells (*PBECs*). 16HBE cells and PBECs were seeded in duplicates, grown to confluence for 3–4 days, serum-deprived (16HBE) or placed on basal medium with transferrin and insulin (PBECs) overnight, pre-treated with or without 0.16-16 nM BUD or 0.1−10 nM FP for 2 hours and stimulated with 5 % CSE and/or 12.5 μg poly-(I:C) for 24 hours. IL-8 was measured in cell-free supernatants. **a** Mean ± SEM IL-8 levels (pg/ml) in 16HBE cells at baseline and upon stimulation with poly-(I:C) in the presence and absence of different concentrations of BUD and FP (*n = 4*). **b** Mean ± SEM IL-8 levels (pg/ml) in 16HBE cells at baseline and upon stimulation cells with 5 % CSE in the presence and absence of 16 nM BUD and 10 nM FP (*n = 4*). **c** Mean ± SEM IL-8 levels (pg/ml) in 16HBE cells upon combined stimulation with CSE + poly-(I:C) in the presence and absence of 16 nM BUD and 10 nM FP (*n = 4*). PBECs were exposed to 12.5 μg/ml poly-(I:C) for 24 hours and absolute IL-8 levels (pg/ml, min to max) (**d**) or values normalized to the absence of BUD or FP (percentage, mean ± SEM) (**e**) are shown (*n* = 6). * = *p* < 0.05 and ** = *p* < 0.01 between the indicated values or between the absence and presence of BUD or FP as determined by the Student’s t-test (assumed normal distribution) (**a**-**c**, **e**) or the Wilcoxon-signed rank test (**d**)

We first assessed the effects of BUD and FP on pro-inflammatory epithelial responses. Exposure to poly-(I:C) for 24 hours strongly increased the secretion of neutrophil chemo-attractant IL-8 (CXCL8), which was completely suppressed by both BUD (0.16, 1.6 and 16 nM) and FP (0.1, 1 and 10 nM, Fig. 1A). Baseline and CSE (5 % for 24 hours)-induced IL-8 levels were also equally efficiently suppressed by BUD (16 nM) and FP (10 nM, Fig. 1B). Furthermore, the combination of CSE (5 %) and poly-(I:C) strongly enhanced IL-8 secretion, which was completely and equally efficiently suppressed by BUD (16 nM) and FP (10 nM, Fig. 1C). Thus, both BUD and FP effectively suppress the production of the pro-inflammatory cytokine IL-8 upon stimulation with CSE and/or poly-(I:C).

To increase the translational relevance, we also studied primary bronchial epithelial cells (PBECs) from smoking individuals using poly-(I:C), the most potent inducer of IL-8 secretion in 16HBE cells. Poly-(I:C) also strongly increased IL-8 secretion in PBECs, which was equally and significantly suppressed by BUD and FP (Fig. 1D,E).

### Effects of BUD and FP on cigarette smoke-induced barrier dysfunction of 16HBE cells and PBECs

**Fig. 2:**
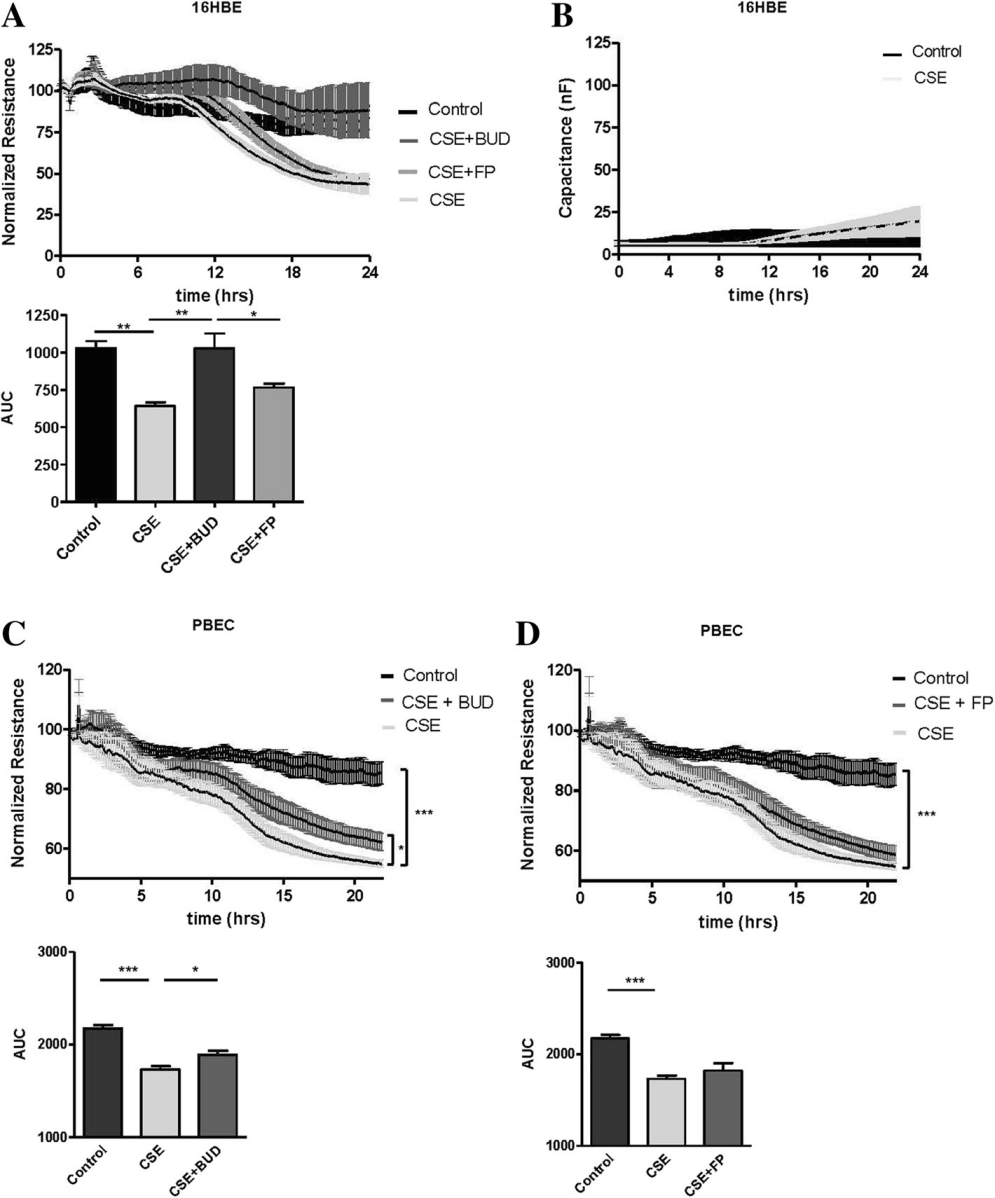
Effect of equivalent concentrations of budesonide (*BUD*) and fluticasone propionate (*FP*) on cigarette smoke extract (*CSE*)-induced barrier dysfunction in 16HBE cells and PBECs. 16HBE cells and PBECs were seeded in duplicates, grown to 80−90 % confluence for 3–4 days, serum-deprived (*16HBE*) or placed on basal medium with transferrin and insulin (*PBECs*) overnight, pre-treated with or without 16 nM BUD or 10 nM FP for 2 hours and exposed to vehicle (control) or 5 % (PBECs) or 7.5 % (16HBE cells) CSE. **a** Electrical resistance in 16HBE cells was measured at 400 Hz using ECIS. Resistance levels were normalized to the levels just prior to the addition of CSE or vehicle. In the upper panel, *p* < 0.01 for control versus CSE, *p* < 0.01 for CSE versus CSE + BUD and *p* < 0.05 for CSE + BUD versus CSE + FP, as analyzed by repeated measures ANOVA. The area-under-the-curve (AUC) was calculated, starting from the time-point of 12 hours after CSE addition, and depicted shown in the lower panel. Mean ± SEM (*n = 6*) levels are shown. * = *p* < 0.05 and ** = *p* < 0.01 between the indicated groups (repeated measures ANOVA with Bonferroni’s post-hoc test). **b** Capacitance in 16HBE cells was measured at 40 kHz during 24 hours using ECIS and mean levels ± SEM are shown (*n = 6*). **c**, **d** Resistance in PBECs was measured at 400 Hz. Resistance levels (Mean ± SEM, *n = 5*) were normalized to the values prior to addition of CSE. The AUC was calculated and levels were analyzed by repeated measures ANOVA (with Bonferroni’s post-hoc test). * = *p* < 0.05, ** = *p* < 0.01 and *** = *p* < 0.001 between the indicated groups

In addition to pro-inflammatory responses, we studied epithelial barrier function using ECIS. The electrical resistance measured by ECIS at a low frequency (i.e. 400 Hz) is highly sensitive to changes in specifically epithelial cell-cell contacts [23, 24]. CSE (7.5 %) induced a marked decrease in electrical resistance (barrier function) of 16HBE cells from 12 hours exposure onwards, with a maximal effect around 24 hours (Fig. 2A). Thereafter, barrier function started to recover slowly (data not shown). CSE did not clearly affect the high-frequency capacitance, a more sensitive parameter to monitor changes in cell-matrix contact (Fig. 2B), indicating that CSE affects epithelial cell-cell contacts rather than cell attachment or viability, in line with our previous observations [12]. When comparing the effects of the equivalent concentrations of 2-hours pre-treatment with 16 nM BUD and 10 nM FP on CSE-induced barrier function in 16HBE cells, FP only modestly, but not significantly attenuated CSE-induced barrier dysfunction, while BUD almost completely prevented the CSE-induced defect in barrier function within 24 hours, the difference between the effect of BUD and FP being statistically significant (Fig. 2A). Similarly to the effects in 16HBE cells, CSE (5 %) markedly reduced electrical resistance in a PBEC monolayer (Fig. 2C). BUD (16 nM) slightly but significantly counteracted the CSE-induced barrier dysfunction, while no significant effect of FP (10 nM) was observed (Fig. 2D). Since the effect of BUD was not as strong as in 16HBE cells, we tested the effect of higher concentrations of BUD and FP (160 nM and 100 nM, respectively). However, neither BUD nor FP exerted a significant effect on CSE-induced barrier dysfunction at this concentration (data not shown). Nevertheless, these results confirm the findings in 16HBE cells, indicating that also in primary epithelial cells BUD and FP differentially affect cigarette-smoke induced barrier dysfunction, while they are equally effective in the inhibition of pro-inflammatory responses.

### Effects of BUD and FP on EGF-induced barrier dysfunction and EGFR downstream signaling

**Fig. 3:**
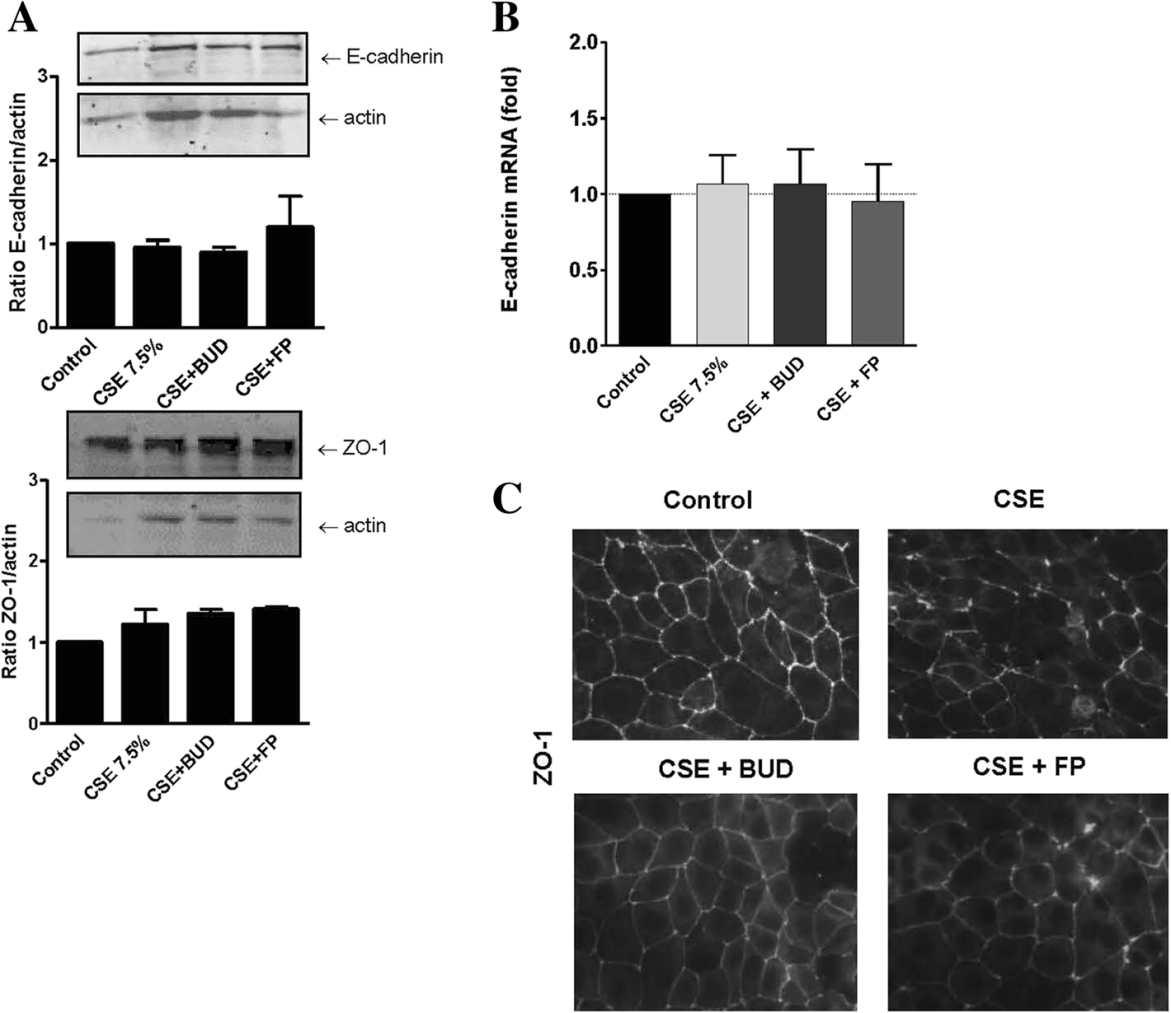
Effect of cigarette smoke extract (*CSE*) and equivalent concentrations of budesonide (*BUD*) and fluticasone propionate (*FP*) on cell-cell contact proteins in 16HBE cells. 16HBE cells were seeded in duplicates, grown to confluence, serum-deprived overnight, pre-treated with or without 16 nM BUD or 10 nM FP for 2 hours and exposed to vehicle (*control*) or 7.5 % CSE (**a**) E-cadherin and ZO-1 protein was detected by western blotting after 24 hours. Actin was used as loading control. Densitometry was performed, protein expression was related to the actin levels and normalized values (Mean ± SEM, *n = 3*) are shown. **b** mRNA was isolated after 6 hours. E-cadherin expression was related to the expression of the housekeeping genes β2μG and PPIA and levels (Mean ± SEM, *n = 4*) are expressed as fold change compared to the control (2^-ΔΔCt^)*.* **c** Immunofluorescent staining for ZO-1 was performed after 24 hours. A representative of 3 independent experiments is shown

**Fig. 4:**
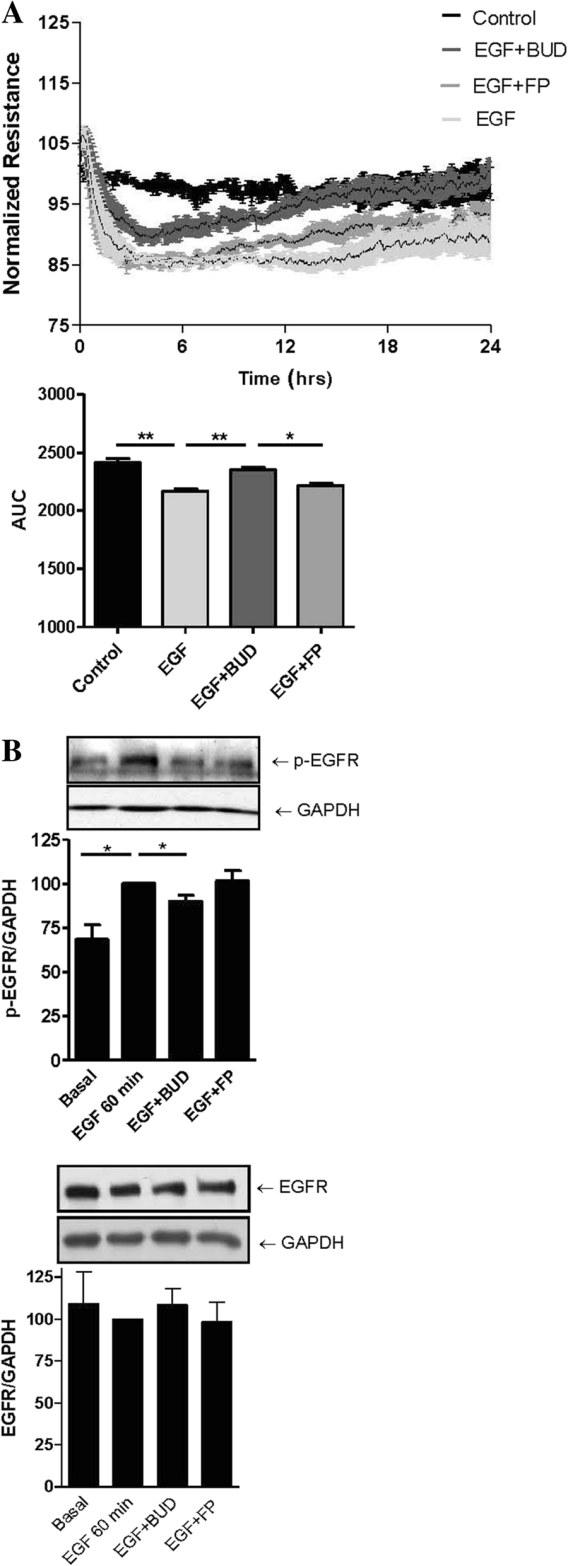
Effect of equivalent concentrations of budesonide (*BUD*) and fluticasone propionate (*FP*) on EGF-induced barrier dysfunction and EGF activity in 16HBE cells. **a** 16HBE cells were seeded in duplicates, grown to confluence, serum-deprived overnight, pre-treated with or without 16 nM BUD or 10 nM FP for 2 hours and exposed to vehicle (*control*) or EGF. Resistance was measured at 400 Hz using ECIS. Resistance levels were normalized to the levels prior to the addition of 7.5 % CSE or vehicle. Mean ± SEM levels (*n = 3*) are shown. In the upper panel, *p* < 0.01 for control versus EGF, *p* < 0.01 for EGF versus EGF + BUD and *p* < 0.01 for EGF + BUD versus EGF + FP, as analyzed by repeated measures ANOVA. The area-under-the-curve (*AUC*) was calculated and mean ± SEM levels (*n = 3*) are shown. * = *p* < 0.05 and ** = *p* < 0.01 between the indicated groups (repeated measures ANOVA with Bonferroni’s post-hoc test). **b** 16HBE cells were grown to confluence, serum-deprived overnight, pre-treated with or without 16 nM BUD or 10 nM FP for 2 hours and exposed to vehicle (*control*) or EGF for 60 min. Phosphorylated EGF receptor (*p-EGFR*) or total EGFR was detected by western blotting. GAPDH was used as loading control. Densitometry was performed, protein expression was related to the actin levels and normalized values (Mean ± SEM, *n = 6*) are shown. * = *p* < 0.05 between the indicated groups as determined by the Student’s *t*-test

To elucidate the mechanism involved in the differential effect of BUD and FP on CSE-induced barrier dysfunction, we first assessed whether the observed effects are exerted on epithelial cell-cell contacts. We studied the expression of the adherens junction protein E-cadherin and the tight junction protein ZO-1 in the presence of CSE. CSE (7.5 % for 24 hours) exposure did not significantly affect total protein expression of ZO-1 and E-cadherin, nor did the addition of BUD or FP (Fig. 3A). Accordingly, mRNA expression of E-cadherin did not change upon CSE (7.5 % for 6 hours) exposure alone or in the presence of BUD and FP (Fig. 3B). On the other hand and in line with our previous observations [12], CSE (7.5 % for 24 hours) disrupted junctional ZO-1 expression as assessed by immunofluorescent staining (Fig. 3C). The presence of either BUD or FP attenuated this effect, while differences between BUD and FP treatment were hardly visible using this semi-quantitative technique. We previously observed that EGFR activation is involved in CSE-induced barrier dysfunction and disruption of ZO-1 expression [12]. Therefore, we also studied the effect of BUD and FP upon exposure to EGF. We observed that BUD, but not FP, significantly counteracted the EGF-induced epithelial barrier dysfunction (Fig. 4A), with also a significant difference between the effect of BUD and FP. Western blotting revealed that EGF-induced EGFR Tyr1174 phosphorylation between 30–120 min (data not shown), with the most pronounced effect at 60 min (Fig. 4B). BUD significantly reduced EGF-induced EGFR phosphorylation, leaving total EGFR levels unaffected, while FP had no effect on phospho-EGFR levels (Fig. 4B). These data suggest that only BUD attenuates EGFR activation and subsequent disruption of cell-cell contacts.

**Fig. 5:**
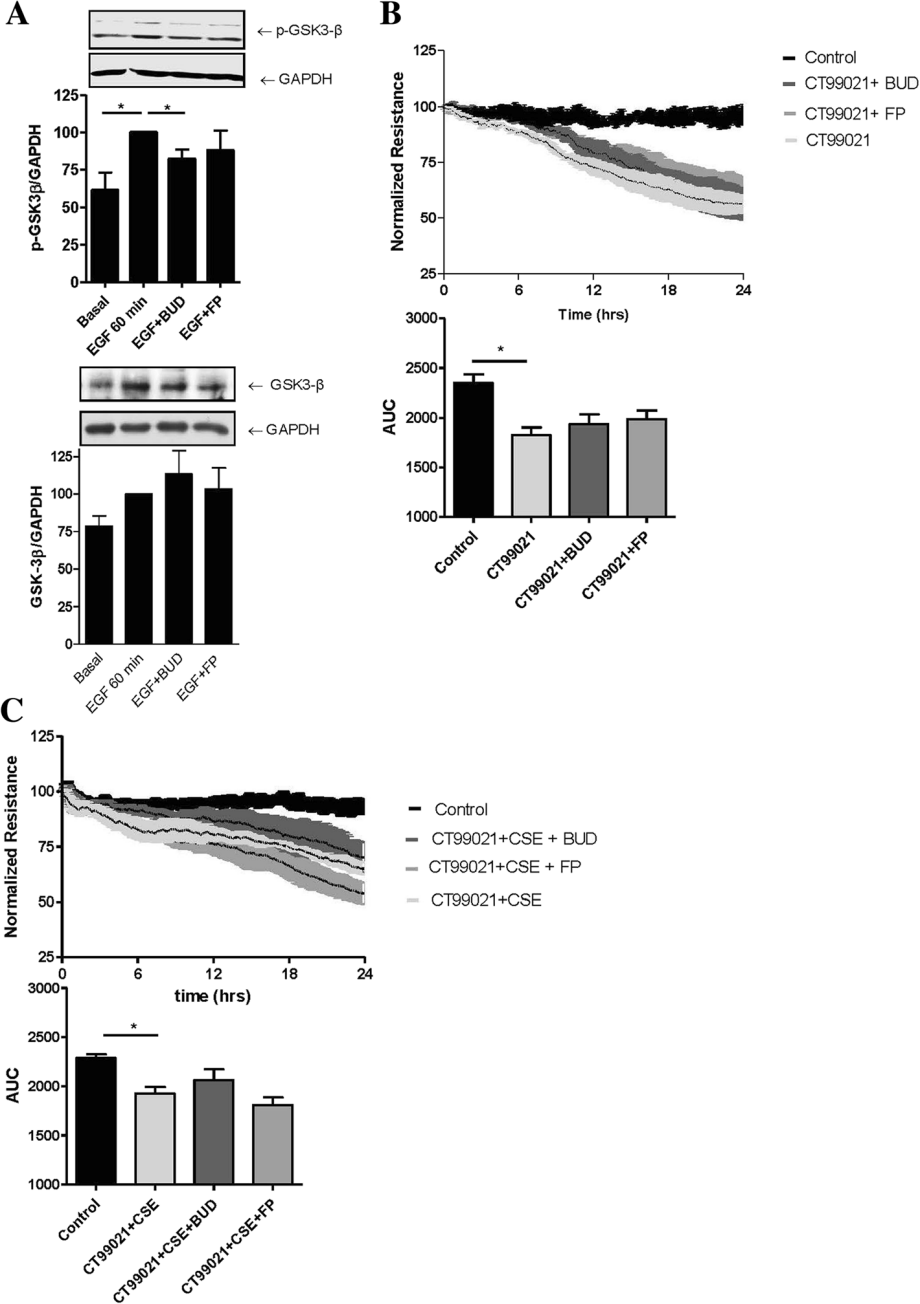
Effect of equivalent concentrations of budesonide (BUD) and fluticasone propionate (FP) on GSK-3β phosphorylation and epithelial barrier dysfunction upon pharmacological inhibition of GSK-3β. **a** 16HBE cells were seeded in duplicates, grown to confluence, serum-deprived overnight, pre-treated with or without 16 nM BUD and 10 nM FP for 60 min and exposed to vehicle (control) or EGF for 60 min. Phosphorylated GSK-3β (*p-GSK-3β*) or total GSK-3β was detected by western blotting. GAPDH was used as loading control. Densitometry was performed, protein expression was related to the actin levels and normalized values (Mean ± SEM, *n = 6*) are shown. * = *p* < 0.05 between the indicated groups. **b**, **c**) 16HBE cells were grown to confluence, serum-deprived overnight, pre-treated with or without 16 nM BUD and 10 nM FP for 2 hours and exposed to vehicle (*control*), GSK-3β inhibitor CT99021 (**b**) or the combination of 7.5 % CSE and CT99021 **c**. Electrical resistance was measured at 400 Hz using ECIS. Resistance levels were normalized to the levels prior to the addition of CSE and/or CT99021. Mean ± SEM levels (*n = 3−4*) are shown. In the upper panels (**b** and **c**), *p* < 0.01 for CT99021 and CT99201 + CSE versus control, as analyzed by repeated measures ANOVA. The area-under-the-curve (AUC) was calculated and mean ± SEM levels (*n = 3−4*) are shown. * = *p* < 0.05 between the indicated groups (repeated measures ANOVA with Bonferroni’s post-hoc test)

Dexamethasone can attenuate the inhibitory phosphorylation of EGFR downstream target GSK-3β [27, 28] . Therefore we assessed whether BUD and FP differently affect the EGF-induced inhibitory phosphorylation of GSK-3β at Ser9. Indeed, EGF significantly increased GSK-3β phosphorylation between 30–120 min (data not shown), with the most pronounced effect at 60 min, which was significantly reduced by BUD, but not FP, while total GSK-3β levels were not affected by BUD or FP (Fig. 5A). Subsequently, we investigated the effects of the pharmacological ATP competitive GSK-3β inhibitor CT99201 on epithelial barrier function. Of note, addition of CT99021 alone induced barrier dysfunction (Fig. 5B), and this effect was not counteracted by either BUD or FP (Fig. 5B). Furthermore, neither BUD nor FP significantly reduced CSE (7.5 %)-induced barrier dysfunction in the presence of CT99021 (Fig. 5C). This indicates that BUD no longer protects against CSE-induced barrier dysfunction when GSK-3β activity is blocked. Together, our results indicate that BUD exerts stronger protective effects than FP on CSE-induced barrier dysfunction in 16HBE cells and PBECs, and that the effect of BUD likely involves the attenuation of EGFR-dependent inactivation of GSK-3β as demonstrated in 16HBE cells.

### Effects of BUD and FP on poly-(I:C)-induced barrier dysfunction of 16HBE cells in the presence and absence of CSE

**Fig. 6:**
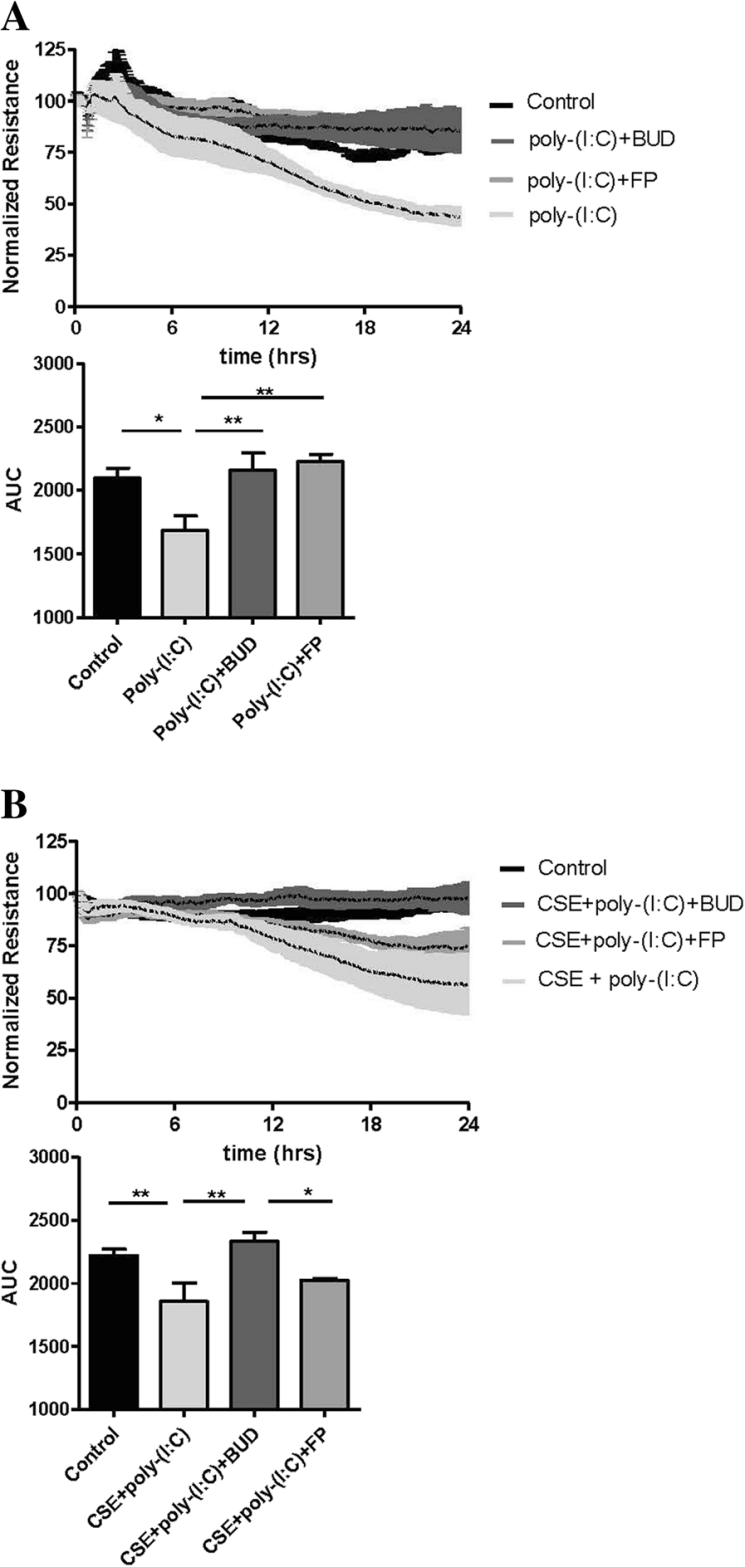
Effect of equivalent concentrations of budesonide (*BUD*) and fluticasone propionate (*FP*) on cigarette smoke extract (*CSE*) and/or poly-(I:C)-induced barrier dysfunction in 16HBE cells. 16HBE cells were seeded in duplicates, grown to confluence, serum-deprived overnight, pre-treated with or without 16 nM BUD or 10 nM FP for 2 hours and exposed to vehicle (control), poly-(I:C) (**a**) or 7.5 % CSE plus poly-(I:C) (**b**). Resistance was measured at 400 Hz using ECIS. Resistance levels were normalized to the levels prior to the addition of poly-(I:C) and mean ± SEM levels (*n = 3−4*) are shown. In the upper panels, *p* < 0.01 for control versus poly-(I:C), p < 0.05 for poly-(I:C) versus poly-(I:C) + BUD and poly-(I:C) + FP (**a**), *p* = 0.05 for control versus CSE + poly-(I:C), *p* < 0.05 for CSE + poly-(I:C) versus CSE + poly-(I:C) + BUD and p < 0.001 for CSE + poly-(I:C) + BUD versus CSE + poly-(I:C) + FP (**b**), as analyzed by repeated measures ANOVA. The area-under-the-curve (AUC) was calculated and mean ± SEM levels (*n = 3−4*) are shown. * = *p* < 0.05 and ** = *p* < 0.01 between the indicated groups (repeated measures ANOVA with Bonferroni’s post-hoc test)

Additionally, we studied whether BUD and FP also differently affect epithelial barrier function upon a different insult, i.e. viral mimetic poly-(I:C). Similar to the effects of CSE and EGF, poly-(I:C) (12.5 μg/ml) markedly reduced epithelial resistance (400 Hz, Fig. 6A), the effect being most pronounced between 12–48 hours. In contrast to the differential effects of BUD and FP on CSE-induced barrier dysfunction, both BUD (16 nM) and FP (10 nM) significantly counteracted poly-(I:C)-induced epithelial barrier dysfunction. As for the involved mechanism, Rezaee et al. previously reported that poly-(I:C) disrupts epithelial integrity at 24 hours by a protein kinase D (PKD)-dependent mechanism [29] . However, we observed that the early poly-(I:C)-induced disruption of 16HBE cell-cell interactions, as measured by low-frequency resistance, could not be blocked by 5 μM PKD inhibitor Gö6976 (data now shown).

Next, we investigated the effect of BUD and FP upon barrier dysfunction induced by the combination of poly-(I:C) and 7.5 % CSE (Fig. 6B). Of note, treatment with BUD protected significantly against the defect in barrier function induced by the combination of CSE and poly-(I:C), while the smaller effect of FP was not statistically significant (Fig. 6B) and the difference between the effect of BUD and FP was significant. Thus, whereas BUD and FP are equally effective in the protection against poly-(I:C)-induced barrier dysfunction, only BUD efficiently counteracts the barrier dysfunction induced by CSE alone or in concerted action with poly-(I:C).

**Fig. 7:**
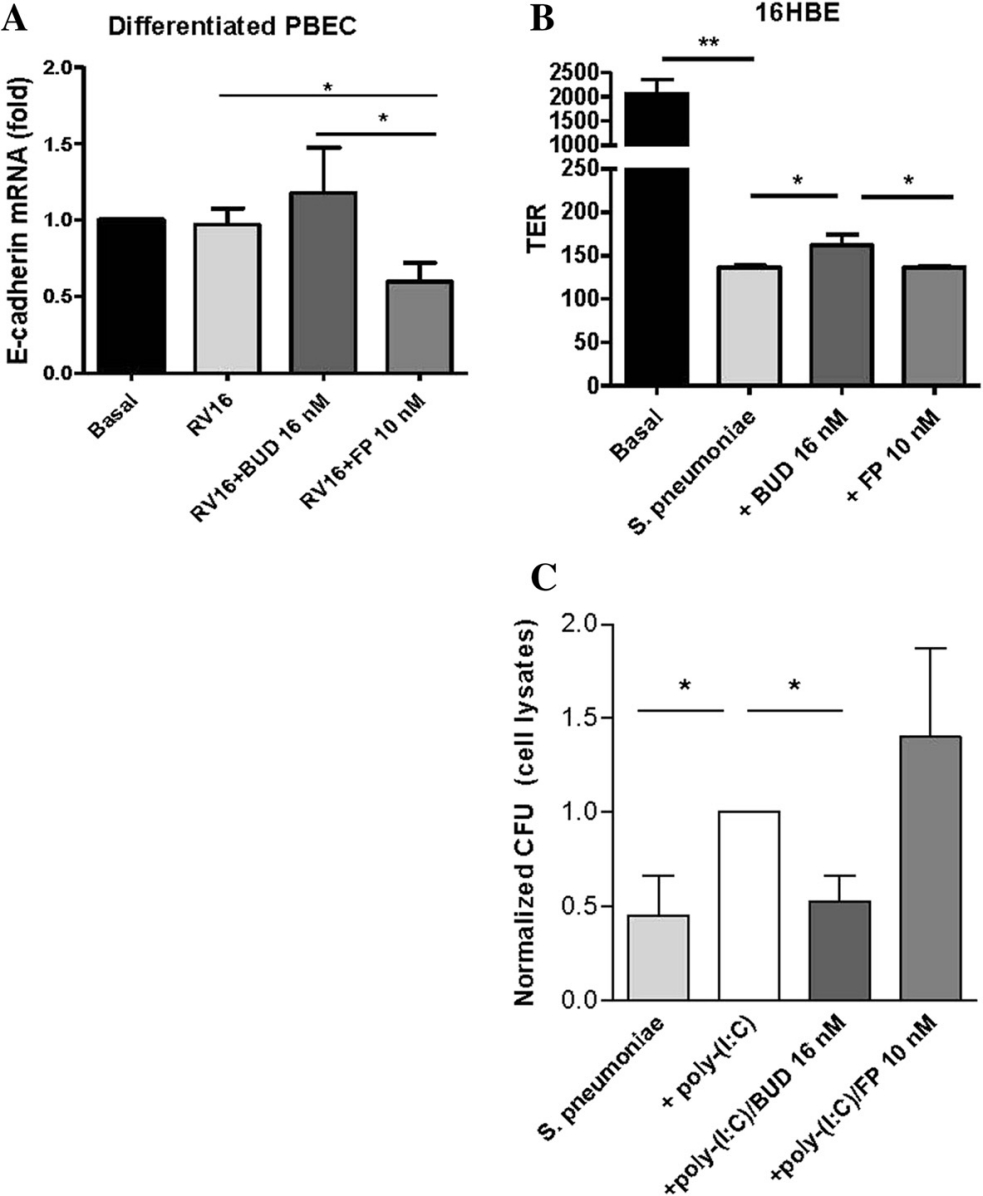
Effects of equivalent concentrations of budesonide (*BUD*) and fluticasone propionate (*FP*) pre-treatment on rhinovirus (*RV16*)-exposed primary mucociliary epithelium and *Streptococcus pneumonia*-exposed 16HBE cells. **a** PBECs were seeded in duplicates in the apical compartment of a transwell system, grown to confluence, differentiated at air-liquid interface for 4 weeks, placed in hormonally-deprived medium overnight, pre-treated with or without 16 nM BUD or 10 nM FP for 2 hours and infected with 50 μl RV16 with an multiplicity of infection of 1 for 24 hours at 37 °C. E-cadherin expression was related to the expression of the housekeeping genes β2μG and PPIA and levels (Mean ± SEM, *n = 10*) are expressed as fold change compared to the control (2^-ΔΔCt^)*.* **b** 16HBE cells were seeded in duplicates in the apical compartment of a transwell system, grown to confluence, serum-deprived overnight, pre-treated with or without 16 nM BUD or 10 nM FP for 2 hours and exposed to *Streptococcus pneumoniae* in the presence and absence of poly-(I:C) for 24 hours. Transepithelial resistance (TER) levels (Ω, mean ± SEM, *n = 7*) (**c**) Colony forming units (CFUs) in 16HBE cell lysates. Invasion in the presence of poly-(I:C) was set at 1 and normalized values are shown (mean ± SEM, *n = 7*). * = *p* < 0.05 and *** = *p* < 0.001 between the indicated groups as determined by the Wilcoxon-signed rank test

To enhance the relevance of our findings, we aimed to study the effects of BUD and FP on live virus-induced barrier dysfunction in ALI-differentiated mucociliary epithelium, reflecting the epithelial layer in vivo more closely. We exposed the cells to RV16, which was previously shown to reduce barrier function in 16HBE cells [13]. However, we were unable to observe effects on epithelial barrier function upon exposure to RV16. Epithelial resistance levels dropped considerably as soon as vehicle was added to the apical site of the ALI culture and the cells were no longer air-exposed, and then an additional effect of RV16 exposure was not observed (data not shown). Furthermore, RV16 exposure for 24 hours did not alter mRNA expression of E-cadherin, nor did pre-treatment with BUD. In contrast, pre-treatment with FP significantly reduced E-cadherin in RV16-exposed epithelium, with a significant difference between BUD and FP (Fig. 7A). Treatment with FP, but not BUD, may thus lead to deterioration of epithelial barrier dysfunction upon viral infection.

Finally, we assessed the effect of live bacteria on barrier function, and studied whether exposure to poly-(I:C) enhances bacterial adhesion/internalization and/or transmigration across the cell interior. The infection of 16HBE cells with *S. pneumoniae* caused a strong decrease in transepithelial resistance, on which BUD, but not FP, exerted a modest but significant protective effect (Fig. 7B). Within the time frame of our experiment, the concomitant exposure to the bacteria and poly-(I:C) did not affect epithelial transmigration of bacteria (data not shown). However, poly-(I:C) strongly increased CFUs in the cell lysates, indicating that poly-(I:C) promotes bacterial adhesion and/or internalization (Fig. 7C). This effect was prevented by BUD, which may thus act to decrease susceptibility to secondary bacterial infection; in contrast FP had no effect (Fig. 7C).

## Discussion

We hypothesized that BUD is more efficacious than FP in the protection of airway epithelial barrier function upon cigarette smoking or viral infection, potentially contributing to the ICS’ differential risk associated with pneumonia in COPD. Our results show that BUD protects more effectively than FP against CSE-induced bronchial epithelial barrier dysfunction, either alone or in combination with viral mimetic poly-(I:C), while BUD and FP equally effectively protect against poly-(I:C)-induced barrier dysfunction. Furthermore, both BUD and FP strongly suppress CSE and/or poly-(I:C)-induced pro-inflammatory cytokine production in bronchial epithelial cells lines and PBECs of smokers without significant differences between the drugs. Our findings may have important implications, since they help to explain the clinical observations that treatment with BUD is not, or seldom associated with an increased risk to develop pneumonia in patient with COPD, in contrast to FP. The present study suggests that this is unlikely a consequence of increased immunosuppression by FP. Instead, treatment of COPD patients with BUD could provide better protective effects against cigarette smoke-induced epithelial damage than FP, reinforcing the epithelial barrier. This may limit the access of pathogens upon cigarette smoking in the presence or absence of a viral infection in vivo. Indeed, we observed that BUD protected against the poly-(I:C)-induced increase in bacterial adhesion and/or internalization. This effect may be mediated by the reinforcement of the epithelial barrier, as epithelial junctions functionally segregate the basolateral from the apical site. RV-induced disruption of epithelial barrier function may thus increase the exposure of cell surface receptors for bacterial binding [30]. Whether loss of epithelial polarity indeed acts to enhance binding of *S. pneumoniae* to surface receptors will require further investigation. Our findings on the reduced expression of E-cadherin mRNA in RV16-exposed differentiated epithelium upon FP treatment suggest that the treatment with FP, but not BUD, may aggravate RV-induced barrier disruption in vivo, and thus increase the risk of a secondary bacterial infection.

Our data indicate that the differential effects of BUD and FP on epithelial barrier function are due to differences in their effect on specific pathways involved in barrier dysfunction upon CSE exposure. While BUD attenuated the EGF-induced phosphorylation of EGFR and its downstream target GSK-3β, FP was not able to do so. This inhibitory effect of BUD on EGFR signaling could be involved in the protective effect of BUD on CSE-induced barrier dysfunction, since we have previously reported that CSE-induced EGFR phosphorylation results in epithelial barrier dysfunction by delocalization of ZO-1 from tight junctions [12]. CSE has also been described to inhibit GSK-3β activity by its phosphorylation at Ser9 in lung epithelial cells [31]. To our knowledge, we are the first to demonstrate that pharmacological inhibition of GSK-3β results in epithelial barrier dysfunction, suggesting that the attenuation of EGFR-dependent GSK-3β phosphorylation by BUD is involved in the protective effect of BUD on epithelial barrier function. We observed that BUD was not able to restore epithelial barrier dysfunction upon pharmacological inhibition of GSK-3β, indicating that GSK-3β activation is indispensable for the responsiveness of the epithelial barrier to BUD, in line with previous findings in lymphoma cells [32]. GSK-3β can induce degradation of transcriptional repressor Slug/Snail2 [33], leading to upregulation of E-cadherin, ZO-1, claudins and occludin expression [34]. Since E-cadherin mRNA expression was not affected by CSE and corticosteroids in our setting, transcriptional regulation of junctional proteins does not likely contribute to the observed effects on epithelial barrier function. Alternatively, Snail can induce disruption of tight junction complexes at the posttranslational level, by causing alternative splicing of ZO-1, resulting in higher expression of the ZO-1 isoform that is involved in junctional plasticity [34]. Future studies will have to determine whether EGFR-dependent GSK-3β inactivation and subsequent degradation of Snail are involved in CSE-mediated disruption of epithelial junctions. In addition to its effects on barrier function, GSK-3β has been implicated in inflammatory responses to bacterial infection [35]. Furthermore, side-stream cigarette smoke-induced inactivation of GSK-3β was shown to increase the susceptibility to adenovirus by the upregulation of its receptor in airway epithelial cells [36], with additional implications for the susceptibility to microbial infection.

It is not fully clear why BUD is more efficient than FP in suppressing the CSE-induced EGFR/GSK-3β pathway, while these ICS are equally effective in suppressing pro-inflammatory cytokine production and providing protection against barrier dysfunction by viral mimicry. It could be speculated that different pathways are involved in these specific processes, although the pathways involved in poly-(I:C)-induced barrier dysfunction need further investigation. With respect to their physicochemical properties, BUD is less lipophilic than FP and has a higher aqueous solubility, leading to a faster dissolution rate and a shorter retention time in the lining fluid of the airways. By contrast, after absorption from the airway lumen, BUD is retained in the airway epithelium for a longer time than FP [9, 10]. This is due to the conjugation of BUD with endogenous fatty acids, resulting in a very lipophilic ester depot from which BUD is slowly released [9]. This prolongs anti-inflammatory effects of BUD in the airway tissue [37] and may possibly also prolong effects of BUD on specific intracellular pathways. Fatty acid esterification of BUD has also been detected in the human lungs [38]. Further studies are required to elucidate whether BUD and FP exert differential effects on pathways involved in pro-inflammatory and antimicrobial responses to viral infection. Additionally, recent findings in human bronchial epithelial cells show that a given glucocorticosteroid induces a unique gene expression “fingerprint” [39], and this may explain some of the differences observed between BUD and FP in the present study.

## Conclusions

Together, our data show that BUD is more efficient in the protection against cigarette smoke-induced epithelial barrier dysfunction than FP, and suggest that this is due to more efficient suppression of EGFR/GSK-3β signaling. We anticipate that this may have important implications for the reinforcement of airway epithelial barrier function upon cigarette smoking in vivo, where treatment with BUD could provide better protective effects than FP, limiting the access of pathogens.

## Abbreviations

BUD: budesonide
BEGM: bronchial epithelium growth medium
COPD: Chronic Obstructive Pulmonary Disease
ECIS: Electric Cell-surface Impedance Sensing
EGFR: epidermal growth factor receptor
FEV: forced expiratory volume
GSK-3: glycogen synthase kinase-3
ICS: inhaled corticosteroids
FP: fluticasone propionate
PBECs: primary bronchial epithelial cells
ZO-1: zona occludens
